# Effects of resistance training on body composition and physical function in elderly patients with osteosarcopenic obesity: a systematic review and meta-analysis

**DOI:** 10.1007/s11657-022-01120-x

**Published:** 2022-06-03

**Authors:** Jia-ming Yang, Hua Ye, Qiang Zhu, Jia-hong Zhang, Qin-qin Liu, Hui-yong Xie, Yi Long, Hui Huang, Yan-long Niu, Yun Luo, Mao-yuan Wang

**Affiliations:** 1 School of Rehabilitation Medicine, Gannan Medical University, Ganzhou, China; 2 Department of Rehabilitation Medicine, First Affiliated Hospital of Gannan Medical University, 128 Jinling Road, Zhanggong District, Ganzhou City, 341000 Jiangxi Province China; 3 Ganzhou Key Laboratory of Rehabilitation Medicine, First Affiliated Hospital of Gannan Medical University, Ganzhou, China

**Keywords:** Resistance training, Osteosarcopenic obesity, Body composition, Physical function

## Abstract

***Summary*:**

Osteosarcopenic obesity (OSO) is a complex disease commonly seen in the elderly. We found that resistance training may improve bone mineral density, skeletal muscle mass, and body fat percentage in patients with OSO. Therefore, resistance training is beneficial for elderly OSO patients and is worth being promoted.

**Purpose:**

Investigate effects of resistance training on body composition and physical function in elderly osteosarcopenic obesity (OSO) patients.

**Methods:**

PubMed, Web of Science, Embase, Cochrane Library, Medline, SinoMed, CNKI, and Wanfang Database were searched from inception until October 13, 2021.Two independent researchers extracted the key information from each eligible study. The methodological quality of included studies was assessed using the Physiotherapy Evidence Database (PEDro) scale. The Cochrane Risk of Bias Tool was used to assess the risk of bias. Grading of Recommendations Assessment Development and Evaluation (GRADE) was used to evaluate the quality of the outcomes. Sensitivity analysis indicated the stability of the results. Statistical analysis was performed using Review Manager 5.3.

**Results:**

Four randomized controlled studies meeting the inclusion criteria were included, with 182 participants. Twelve weeks of resistance training improved bone mineral density (BMD, mean difference (MD) = 0.01 g/cm^2^, 95% confidence interval (CI): 0.001, 0.02, *P* = 0.03, *I*^2^ = 0%), skeletal muscle mass (SMM, MD = 1.19 kg, 95% CI: 0.50, 1.89, *P* = 0.0007, *I*^2^ = 0%), *Z* score, timed chair rise test (TCR), and body fat percentage (BFP, MD =  − 1.61%, 95% CI: − 2.94, − 0.28, *P* = 0.02, *I*^2^ = 50%) but did not significantly affect skeletal muscle mass index (SMI, MD = 0.20 kg/m^2^, 95% CI: − 0.25, 0.64, *P* = 0.38, *I*^2^ = 0%) or gait speed (GS).

**Conclusions:**

Resistance training is a safe and effective intervention that can improve many parameters, including BFP, SMM, and *Z* score, among OSO patients and is a good option for elderly individuals to improve their physical fitness.

**Supplementary Information:**

The online version contains supplementary material available at 10.1007/s11657-022-01120-x.

## Highlights

1. Osteosarcopenic obesity is a combination of osteoporosis, sarcopenia, and obesity.
2. Osteosarcopenic obesity may lead to higher rates of disability and mortality.
3. Resistance training can improve body composition and physical function.
4. Resistance training is beneficial to elderly osteosarcopenic obesity patients.

**Supplementary Information:**

The online version contains supplementary material available at 10.1007/s11657-022-01120-x.

## Introduction

With the rapid development of the world economy, some countries have become aging societies, and an aging population results in greater health challenges. Aging can lead to some diseases in the elderly, such as sarcopenia [1–4], obesity [5–7], and osteoporosis [8–10]. Sarcopenia can cause muscle weakness [11] and increase the risk of falls and fractures [12]. Obesity is an important cause of metabolic syndrome [13] and chronic diseases such as hypertension [14–16], hyperlipidemia [17], hyperglycemia [18, 19], and cardiovascular diseases [20–23]. Osteoporosis is a risk factor for fragility fracture [24, 25]. The concomitant occurrence of all three diseases is referred to as osteosarcopenic obesity (OSO), which places a very large burden on global health [26]. Studies have shown that OSO is associated with a reduced functionality [27, 28], frailty [29] and falls [27] in patients. In 2014, the concept of OSO was first proposed by Ilich et al. that patients suffer from both bone loss, muscle loss, and fat gain [30]. Since then, OSO has gradually attracted people’s attention, and the corresponding diagnostic criteria [31] and treatment principles [32, 33], including nutritional intervention [34–37] and exercise intervention [38], have been proposed. In 2019, Kelly et al. revised the physical diagnostic criteria for OSO based on the differences in body composition between men and women [39]. However, this diagnostic criterion may only be applicable to European populations and may need to be modified when used to diagnose other ethnic groups.

Exercise, especially resistance training, is considered an effective strategy for the treatment of sarcopenic obesity [40–44] and osteosarcopenia [26, 45]. Sarcopenic obesity is more dangerous than sarcopenia or obesity alone [46] because sarcopenia and obesity may interact with each other to maximize their impact on morbidity, disability, and mortality in older people [46]. And osteosarcopenia is associated with greater health risks than osteoporosis or sarcopenia alone [47]. OSO is a combination of osteoporosis, sarcopenia, and obesity, which may be much more severe than osteoporosis, sarcopenia, or obesity alone [48]. Patients with OSO may have greater health risks than individuals with sarcopenic obesity or osteosarcopenia. As previously mentioned, resistance training is beneficial in improving sarcopenic obesity and osteosarcopenia, so is resistance exercise equally effective for OSO? Currently, only a few small sample clinical studies have been conducted to explore this question but have obtained different results [49–53]. Nevertheless, there has been no systematic review or meta-analysis conducted to evaluate the efficacy of resistance training interventions on OSO.

Consequently, the purpose of this study was to explore the effect of resistance training on parameters related to the physical health and functional performance of elderly patients with OSO, including body composition, physical function, and OSO *Z* score.

## Methods

This article was performed according to the Preferred Reporting Items for Systematic Reviews and Meta-Analyses (PRISMA) guidelines [54]. This article has been registered on PROSPERO, the registration number is CRD42021285205.

### Search strategies

We searched the published literature on PubMed, Medline, Web of Science, Embase, Cochrane Library, SinoMed, CNKI, and Wanfang Database without imposing publication date or language restrictions. A systematic search was performed for eligible studies published through Oct 13, 2021. The keywords were (osteosarcopeni* OR osteo-sarcopeni* OR sarco-osteopeni* OR sarco-osteoporo*) AND (obesity OR adiposity OR overweight) AND (resistance training OR resistance exercise OR strength training) AND (randomized controlled OR RCT OR controlled trial OR clinical trial). The PubMed search strategy is shown in Table S1. In addition, to prevent the omission of relevant studies, a further manual search of references was performed to identify potential studies that were not captured by database retrieval. For example, we searched the references of included studies to prevent the omission of relevant literature.

### Study selection

Two authors (Hua Ye, Jia-hong Zhang) independently selected the literature. If any differences existed, a meeting was held to resolve them. The inclusion criteria for the studies were based on the PICOS (patients, intervention, comparison, outcomes, and study design) principle [55], as shown below:

- **Patients (P):** People with OSO (participants were required to have a combination of osteopenia, sarcopenia, and obesity and to have no other diseases, such as fractures, heart failure, and diabetes). Participants were required to be elderly (age ≥ 60), but there were no restrictions for sex or environment (such as hospitals, communities, or nursing homes).
- **Intervention (I):** Interventions included resistance training, such as elastic resistance training and progressive resistance training.
- **Comparison (C):** Control group or placebo.
- **Outcomes (O):** Primary outcomes: body composition (e.g., body fat percentage (BFP), skeletal muscle mass index (SMI), bone mineral density (BMD)); secondary outcomes: physical function (e.g., hand grip strength (HGS), gait speed (GS)) and OSO *Z* score. Among them, BFP, SMI, and BMD were calculated using data obtained from the dual-energy X-ray absorptiometry (DXA). The HGS of the subject’ s dominant hand was measured using a standard hydraulic hand dynamometer. A 10-m walk test (10 MWT) was measured to obtain the GS of the participants.
- **Study design (S):** Only randomized controlled trials were included in this study.

For articles with overlapping data of the same population source, only the largest report was included, unless they reported different outcomes of interest.

Studies were excluded if they met the following criteria: (1) participants were not patients with OSO (for example, patients with just osteoporosis, sarcopenia or obesity, or with other diseases, such as fractures or heart failure); (2) interventions did not include resistance training; (3) the comparisons were not performed according to the intervention type; (4) outcomes did not include body composition, physical function, or OSO *Z* score; and (5) the type of studies were not randomized controlled trials.

### Data extraction

Two authors (Qin-qin Liu, Qiang Zhu) independently extracted the data from the selected studies into a Microsoft Excel spreadsheet and then summarized the data in a table. Any disagreement was resolved in a consensus meeting.

The data extracted included (1) first author and year of publication; (2) country or region of author; (3) sex and age of participants; (4) groups and sample size; (5) time points of outcome assessment; (6) total intervention time; (7) outcome indicators; and (8) Physiotherapy Evidence Database (PEDro) scores for each study (as shown in Table 1). In addition, we collected information on the measurement tools used to assess body composition and diagnostic criteria of OSO for each study (as shown in Table 2).

**Table 1:** Characteristics of the included randomized controlled trials

Study	Country/region	Sex, age	Groups (sample size)	Time points of assessment	Duration of intervention	Primary outcomes	Secondary outcomes	PEDro Score
Cunha et al. [50]	Brazil	Women, ≥ 60	G1S (21), G3S (20), CG (21)	Weeks 1 ~ 2, Weeks 15 ~ 16	12 weeks	SMM↑, BFP↓, BMD	OSO Z score↑	7
Banitalebi et al. [53]	Iran	Women, 65 ~ 80	EBRT (32), CG (31)	Baseline, Week 12	12 weeks	BFP, BMD	OSO Z score↑, HGS↑, GS, TUG, TCR↑	7
Lee et al. [51]	Taiwan, China	Women, 60 ~ 90	peRET (15), CG (12)	Baseline, Week 12	12 weeks	BFP, SMM, SMI, BMD	HGS, GS, TUG↑, TCR↑	8
Li et al. [52]	China	Both, > 60	AE + RT (15), CG (15)	Baseline, Week 12	12 weeks	BMD↑, BFP↓, SMI		5

↑ compared with the control group, the end point value increased (*P* < 0.05); ↓ compared with the control group, the end point value decreased (*P* < 0.05)

*PEDro*, Physiotherapy Evidence Database; *G1S*, 1-set group; *G3S*, 3-set group; *CG*, control group; *SMM*, skeletal muscle mass; *BFP*, body fat percentage; *BMD*, bone mineral density; *EBRT*, elastic band resistance training; *OSO*, osteosarcopenic obesity; *HGS*, hand grip strength; *GS*, gait speed; *TUG*, timed up and go test; *TCR*, timed chair rise test; *peRET*, progressive elastic band resistance exercise training; *SMI*, skeletal muscle mass index; *AE*, aerobic exercise; *RT*, resistance training

**Table 2:** Diagnostic criteria for OSO in the included studies

Study	Body composition assessment tool	Diagnostic criteria for osteopenia	Diagnostic criteria for sarcopenia	Diagnostic criteria for obesity
Banitalebi et al. [53]	DXA	− 2.5 ≤ T-score ≤ − 1.0 of L1-L4, and/or total femur or femoral neck	10 MWT ≤ 1 (m/s^2^), and SMI ≤ 28% or ≤ 7.76 kg/m.^2^	BFP > 32%, BMI > 30 kg/m.^2^
Lee et al. [51]	DXA, BIA	T-score < − 1.0 of L1-L4	SMI (appendicular lean mass/height^2^) < 5.67 kg/m.^2^ and a grip strength of < 20 kg or gait speed of < 0.8 m/s	BFP > 35%
Li et al. [52]	DXA, BIA	T-score < − 1.0	Men: SMI ≤ 7.0 kg/m^2^; Women: SMI ≤ 5.4 kg/m.^2^	Men: BFP > 25%; Women: BFP > 35%
Cunha et al. [50]	DXA	OSO diagnostic criteria were not mentioned in the original article		

*OSO*, osteosarcopenic obesity; *DXA*, dual X-ray absorptiometry; *10 MWT*, 10-m walk test; *BFP*, body fat percentage; *SMI*, skeletal muscle mass index; *BMI*, body mass index; *BIA*, bioelectrical impedance analysis

### Quality assessment

Two authors (Hui Huang, Hui-yong Xie) independently completed an assessment of the methodological quality of each included study using the PEDro scale. If the score was inconsistent between the two authors, a third author (Yi Long) was consulted to judge the final score. The PEDro scale has 11 items: (1) eligibility criteria and source, (2) random allocation, (3) concealed allocation, (4) baseline comparability, (5) participant blinding, (6) therapist blinding, (7) assessor blinding, (8) adequate follow-up (> 85%), (9) intention-to-treat analysis, (10) between-group statistical comparisons, and (11) point and variability measurements. A total PEDro score is achieved by adding the ratings of items (2) to (11) for a combined total score between 0 and 10. Scores of less than 4 are considered poor, 4 to 5 are considered fair, 6 to 8 are considered good, and 9 to 10 are considered excellent [56].

In addition, we assessed the quality of each outcome according to the Grading of Recommendations Assessment Development and Evaluation (GRADE) criteria [57] by assigning ratings such as “very low,” “low,” “moderate,” and “high,” which involved evaluating eight domains, including risk of bias, directness of evidence, consistency and precision of results, publication bias, magnitude of effect, dose–response, and influence of confounding factors.

### Risk of bias

Two authors (Hui Huang, Hui-yong Xie) assessed the risk of bias of the included studies using the Cochrane Risk of Bias Tool [58]. If there was a disagreement, a third author (Yi Long) was involved to reach a consensus. There are seven items in the bias risk table: (1) random sequence generation (selection bias); (2) allocation concealment (selection bias); (3) blinding of participants and personnel (performance bias); (4) blinding of outcome assessment (detection bias); (5) incomplete outcome data (attrition bias); (6) selective reporting (reporting bias); and (7) other bias. Each item was classified as low risk, high risk (not fulfilling the criteria), or unclear risk (specific details or descriptions were not reported). Furthermore, the presence of publication bias was estimated using a funnel plot.

### Data synthesis and statistical analyses

Statistical analysis was performed using Review Manager 5.3 (The Nordic Cochrane Centre, Copenhagen, Denmark). The pre-extracted mean value, standard deviation, and sample size were input into the statistical software. In our meta-analyses, we reported the effect size using the mean difference (MD) with 95% confidence interval (95% CI) for studies that used the same measurement methods and the standardized mean difference (SMD) for those that measured the same outcome with different units for continuous outcomes. In all analyses, *I*^2^ statistics were used to analyze heterogeneity between studies. If the *P* value of the heterogeneity test (*I*^2^ statistic) was < 0.05, the random-effects model was used; otherwise, a fixed-effects model was used. For small sample studies and highly heterogeneous outcomes, sensitivity analysis was used to test the stability of the results.

## Results

### Study selection

**Fig. 1:**
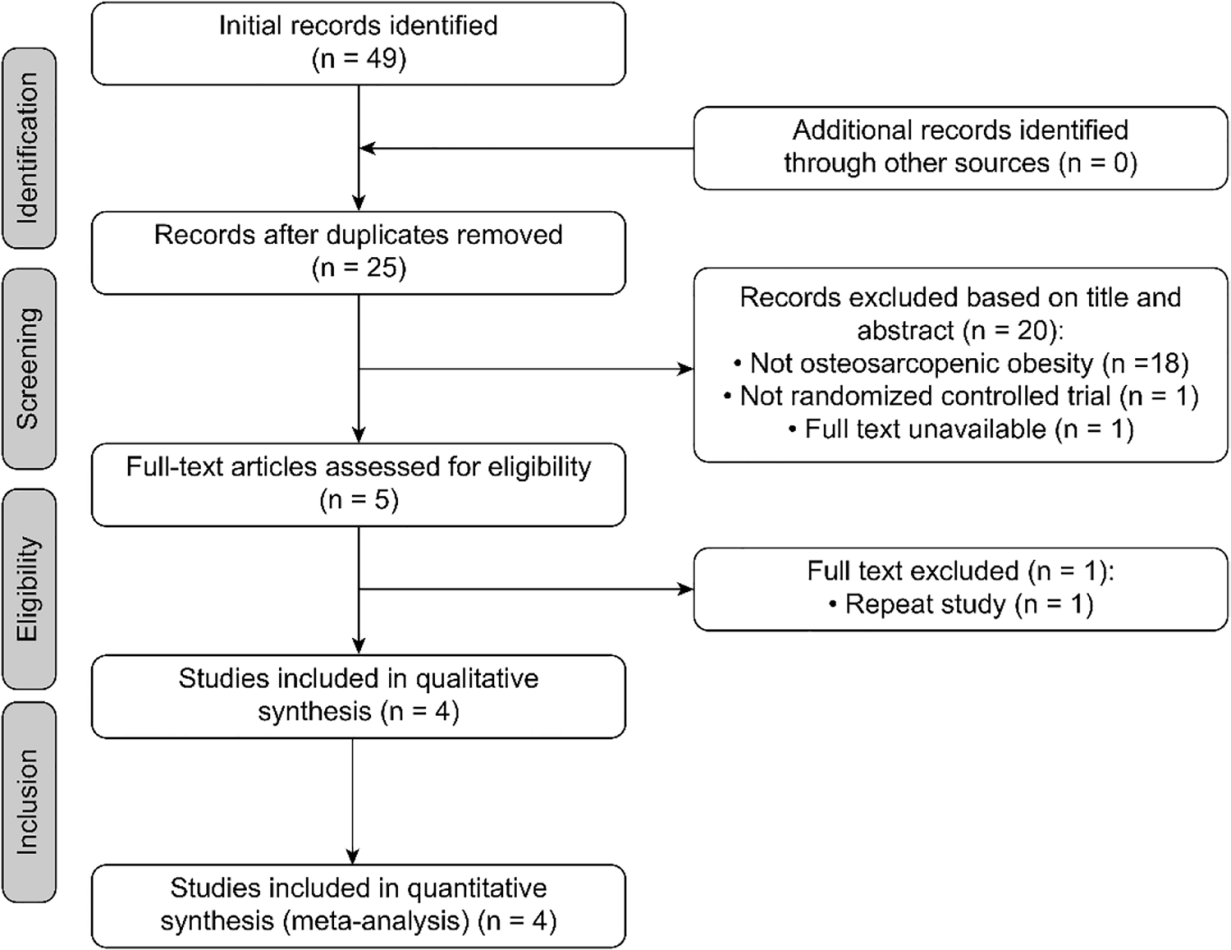
The study selection process

After a systematic search of eight databases, we identified a total of 49 articles, including 7 in PubMed, 19 in Medline, 2 in Embase, 3 in Web of Science, 8 in Cochrane Library, 5 in CNKI, 4 in Wanfang Database, and 1 in SinoMed. No literature was obtained from other sources. After deleting duplicate studies, 25 studies remained. After reading the titles and abstracts, 18 articles were excluded because they were not studies on OSO, 1 study was not an RCT, and the other one was not available, leaving 5 articles. Then, we read the full text of the 5 articles. We noticed that there were two studies that had the same population source and with repeated data on body composition [49, 53]. In addition, Banitalebi et al. (2020) [53] also included physical function indicators, so we included the study with more complete data [53]. The list of literatures exclusion and reasons for exclusion are shown in Table S2. As a result, four studies were included for qualitative analysis [50–53]. Hence, we finally included only four studies in the meta-analysis [50–53] (Fig. 1).

### Study characteristics

Among the four included studies, three studies involved only older women [50, 51, 53], and the other study included both elderly men and women [52]. One study divided resistance training into two groups based on exercise intensity: G1S (participants only performed 1 set of 10–15 repetitions maximum for each exercise) and G3S (participants performed 3 sets of 10–15 repetitions maximum for each exercise) [50]. In three studies, resistance training was conducted by using elastic bands [51–53]. In the remaining study, aerobic exercise was combined with resistance training in the intervention group of patients with OSO [52]. In one study, parameter evaluation was performed in weeks 1–2 and 15–16 for baseline and postintervention [50], and the assessments in the other studies were performed at baseline and postintervention [51–53]. The total intervention duration in all four studies was 12 weeks, and resistance training was performed three times every week.

Regarding the assessment tool of body composition, two studies used only DXA to assess body composition [50, 53], and two studies used both DXA and bioelectrical impedance analysis (BIA) [51, 52]. The diagnostic criteria for OSO were divided into three components: osteopenia, sarcopenia, and obesity. Among them, the diagnostic criteria for osteopenia, sarcopenia, and obesity in one study were as follows: (1) osteopenia: − 2.5 ≤ *T*-score ≤  − 1.0 of L1–L4 and/or total femur or femoral neck; (2) sarcopenia: 10 MWT ≤ 1 (m/s^2^), and SMI ≤ 28% or ≤ 7.76 kg/m^2^; and (3) obesity: BFP > 32%, body mass index (BMI) > 30 kg/m^2^ [53]. One study used a *T*-score <  − 1.0 of L1–L4 to diagnose osteopenia; SMI < 5.67 kg/m^2^ and a grip strength of < 20 kg or gait speed of < 0.8 m/s to diagnose sarcopenia; and BFP > 35% to diagnose obesity [51]. In the other study, the inclusion criteria were (1) osteopenia: *T*-score <  − 1.0; (2) sarcopenia: men: SMI ≤ 7.0 kg/m^2^, women: SMI ≤ 5.4 kg/m^2^; and (3) obesity: men: BFP > 25%, women: BFP > 35% [52]. One study did not mention the diagnostic criteria for OSO in the original text [50] (Table 2).

All the authors of the included studies declared that there was no conflict of interest. One study was funded by the Shahrekord University (grant no. 96INT8M895) [53], and the other was supported by the National Science Council of Taiwan (grant no. NSC 102–2314-B-038–053-MY3) and Taipei Medical University-Wan Fang Hospital, Taiwan (grant no. 98TMU-WFH-05–3) [51].

### Resistance training protocol

Three studies used elastic bands for resistance training [51–53], and one study combined aerobic exercise with resistance training [52]. The resistance training sessions lasted 60 min in one study [53], 40 min in one study [51], and 45 ~ 60 min in the other study [52]. In addition, in another study, the timing of the intervention was related to grouping: the duration of training for the G1S group was 30 min, while that for the G3S group was 50 min [50]. In all four studies, resistance exercise interventions were performed three times per week (such as on Mondays, Wednesdays, and Fridays) for 12 weeks (Table S3).

### Quality of included studies

The PEDro scores for each included study are shown in Table 1, and details of the scores are shown in Table S4. The four studies scored between 5 and 8, with a mean and standard deviation of 6.75 ± 1.258. Only one of the studies scored 5, and the quality of the literature was considered fair [52], while the remaining three scored 7 or 8 and were considered good [50, 51, 53]. All of the included studies had baseline comparability, between-group statistical comparisons, and point and variability measurements. In addition, concealed allocation was used in three studies [50, 51, 53], assessor blinding was used in three studies [50, 51, 53], follow-up rates were greater than 85% in three studies [50–52], and intention-to-treat analysis was used in two studies [51, 53]. One study did not mention the use of blinding in the original text [52].

### Risk of bias of included studies

**Fig. 2:**
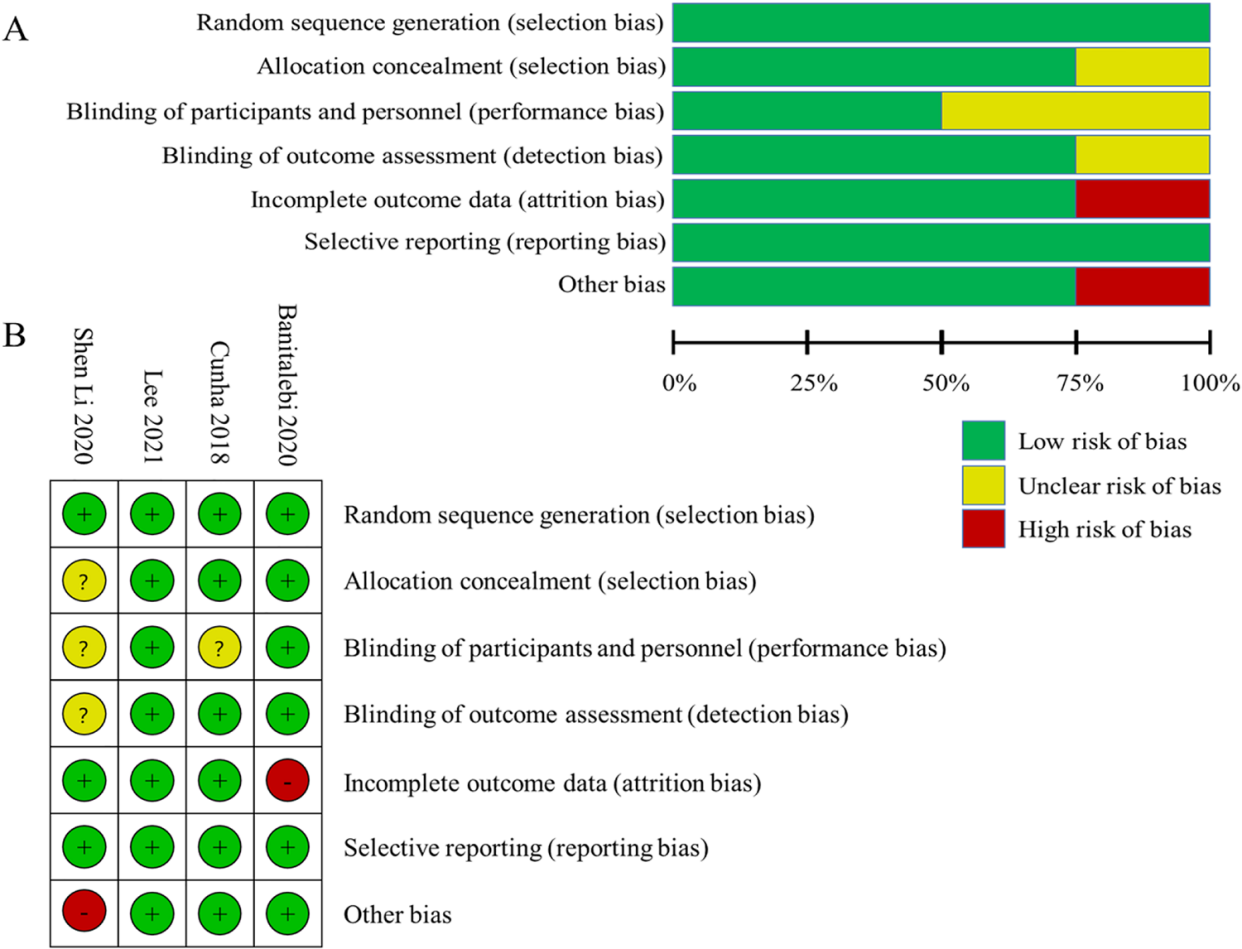
Risk of bias graph and summary of included studies. **A** The risk of bias graph shows the overall risk of bias in each domain. **B** The risk of bias summary indicates the risk of bias in each domain for each study

The risk of bias assessment for four studies is shown in Fig. 2 according to the Cochrane tool. One study did not mention allocation concealment (unclear risk of bias) [52]. Neither participants nor researchers were blinded due to the nature of the intervention in two studies (low risk of bias) [51, 53], while two studies did not mention participants or researchers blinding (unclear risk of bias) [50, 52]. One study had incomplete outcome data because the follow-up rates were lower than 85% (high risk of bias) [53]. One study used not only resistance training but also aerobic exercise as an intervention [52], which may have an impact on the final results and result in a risk of bias (high risk of bias). No studies had selection bias or reporting bias. Due to the insufficient number of included studies, a funnel plot analysis was not performed.

### Effects of resistance training on primary outcomes

Four studies (*n* = 182) investigated the effects of resistance exercise on BMD and BFP [50–53]. Two studies (*n* = 79) assessed skeletal muscle mass (SMM) [50, 51], and two studies (*n* = 57) measured SMI [51, 52].

**Fig. 3:**
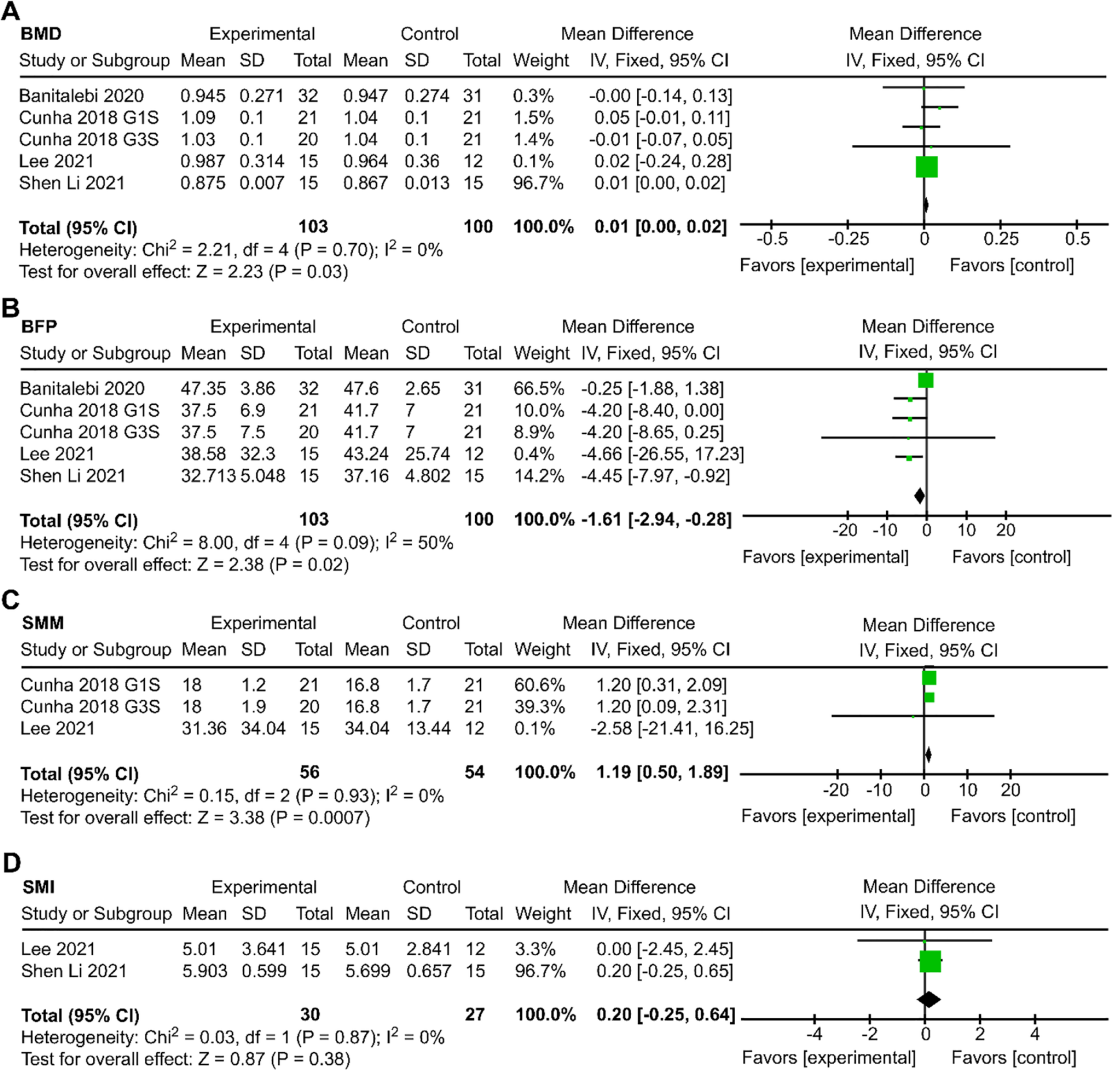
Forest plots of the effect of resistance training compared to that of the control condition on body composition in SO patients. Abbreviations: BMD, bone mineral density; BFP, body fat percentage; SMM, skeletal muscle mass; SMI, skeletal muscle mass index

Forest plot results showed that resistance training can effectively increase BMD (MD = 0.01 g/cm^2^, 95% CI: 0.001, 0.02, *P* = 0.03, *I*^2^ = 0%), significantly increase SMM (MD = 1.19 kg, 95% CI: 0.50, 1.89, *P* = 0.0007, *I*^2^ = 0%), and decrease BFP (MD =  − 1.61%, 95% CI: − 2.94, − 0.28, *P* = 0.02, *I*^2^ = 50%) but that it has no effect on SMI (MD = 0.20 kg/m^2^, 95% CI: − 0.25, 0.64, *P* = 0.38, *I*^2^ = 0%) (Fig. 3).

### Effects of resistance training on secondary outcomes

Because only one study could extract available data for secondary outcomes, it was not possible to conduct a meta-analysis. As shown in Table 1, two studies explored the OSO *Z* score, and the results showed that resistance training can effectively improve the *Z* score [50, 53]. They treated the OSO *Z* score as a composite *Z* score derived from the average of the components calculated based on the following formula: *Z* score = ((muscular strength *Z* score) + (SMM *Z* score) + (− 1 × body fat *Z* score) + (BMD *Z* score))/4 [50, 53]. Two studies measured HGS, GS, timed up and go (TUG), and 30-s timed chair rise (TCR) repetition and showed that 12 weeks of elastic band resistance training significantly improved TCR but had no effect on GS [51, 53]. However, the two studies showed opposite results for HGS and TUG. Banitalebi et al.[53] found that 12 weeks of resistance training increased HGS (*P* = 0.013) but had no effect on TUG (*P* = 0.225). Nevertheless, Lee et al. [51] showed the opposite result: progressive elastic band resistance exercise training could not improve HGS (*P* = 0.413) but could increase TUG (P = 0.030).

### Quality of outcome indicators

The quality of the evidence as assessed using GRADE was rated from very low to high. All outcomes were imprecise because the sample sizes included in the studies were too small. The BMD results may have a serious risk of bias because the intervention in the study by Li et al. [52] was resistance training combined with aerobic exercise, and these results were heavily weighted in the BMD analysis. BFP, HGS, and TUG may have serious inconsistencies because of opposite results or high heterogeneity across studies. As a result, for primary outcomes, the quality of evidence for SMI and SMM was moderate, while that of BMD and BFP was low. Regarding secondary outcomes, the quality of evidence for GS, TCR, and OSO *Z* score was moderate, but that for HGS and TUG was low (Table 3).

**Table 3:** GRADE evidence profile for primary outcomes and secondary outcomes among trials included in the systematic review

No. of studies	Design	Risk of bias	Inconsistency	Indirectness	Imprecision	Other considerations	Resistance training (*n*)	Control (*n*)	Relative effect (95% CI)	Absolute effect	Quality	Importance
Bone mineral density (mean follow-up 12 weeks; measured with dual X-ray absorptiometry; lower values are better)												
4	Randomized trials	Serious*	No serious inconsistency	No serious indirectness	Serious^†^	None	103	100	-	MD 0.01 higher (0 to 0.02 higher)	+ + Low	Critical
Body fat percentage (mean follow-up 12 weeks; measured with dual X-ray absorptiometry; lower values are better)												
4	Randomized trials	No serious risk of bias	Serious^‡^	No serious indirectness	Serious^†^	None	103	100	-	MD 1.61 lower (0 higher to 0.28 lower)	+ + Low	Critical
Skeletal muscle mass (mean follow-up 12 weeks; measured with dual X-ray absorptiometry, bioelectrical impedance analysis; lower values are better)												
2	Randomized trials	No serious risk of bias	No serious inconsistency	No serious indirectness	Serious^†^	None	56	54	-	MD 1.19 higher (0.5 to 1.89 higher)	+ + + Moderate	Important
Skeletal muscle mass index (mean follow-up 12 weeks; measured with: dual X-ray absorptiometry; lower values are better)												
2	Randomized trials	No serious risk of bias	No serious inconsistency	No serious indirectness	Serious^†^	None	30	27	-	MD 0.2 higher (0.25 lower to 0.64 higher)	+ + + Moderate	Critical
Hand grip strength^§^ (mean follow-up 12 weeks; measured with standard hydraulic hand dynamometer; lower values are better)												
2	Randomized trials	No serious risk of bias	Serious^||^	No serious indirectness	Serious^†^	None	47	43	-^§^	Not pooled^§^	+ + Low	Important
Gait speed^§^ (mean follow-up 12 weeks; measured with 10-m walk test; lower values are better)												
2	Randomized trials	No serious risk of bias	No serious inconsistency	No serious indirectness	Serious^†^	None	47	43	-^§^	Not pooled^§^	+ + + Moderate	Important
Timed up and go test^§^ (mean follow-up 12 weeks; lower values are better)												
2	Randomized trials	No serious risk of bias	Serious^||^	No serious indirectness	Serious^†^	None	47	43	-^§^	Not pooled^§^	+ + Low	Important
Timed chair rise test^§^ (mean follow-up 12 weeks; lower values are better)												
2	Randomized trials	No serious risk of bias	No serious inconsistency	No serious indirectness	Serious^†^	None	47	43	-^§^	Not pooled^§^	+ + + Moderate	Important
Osteosarcopenic obesity *Z*-score^§^ (mean follow-up 12 weeks; lower values are better)												
2	Randomized trials	No serious risk of bias	No serious inconsistency	No serious indirectness	Serious^†^	None	73	52	-^§^	Not pooled^§^	+ + + Moderate	Important

^*^The intervention method from Li et al. [52] was resistance training combined with aerobic exercise, which was heavily weighted in bone mineral density

^†^ The sample size was too small (*n* < 400)

^‡^ Moderate heterogeneity (*I*^2^ = 50%)

^§^ Data could not be extracted and merged

^||^ The two studies showed opposite results

### Sensitivity analysis

To test the stability of the meta-analysis results, we performed a sensitivity analysis on the primary outcomes. As seen in the forest plot, the study of Li et al. [52] accounted for 96.7% of the weight in BMD, and we found that the BMD result became meaningless when the study was removed (MD = 0.02 g/cm^2^, 95% CI: − 0.02, 0.06, *P* = 0.37, *I*^2^ = 0%). For BFP, when the study of Banitalebi et al. [53] was removed, the heterogeneity became 0%, and the results became more significant (MD =  − 4.31%, 95% CI: − 6.61, − 2.01, *P* = 0.0002, *I*^2^ = 0%).

## Discussion

In this systematic review and meta-analysis, we included only randomized controlled studies. The purpose of this study was to investigate the effects of resistance training on body composition and physical function in elderly OSO patients. We found that 12 weeks of elastic band resistance training can effectively increase BMD, significantly improve SMM, and distinctly reduce BFP in older people with OSO but had no effect on SMI. In addition, resistance training effectively improved OSO *Z* score and TCR but not GS. Regarding the influence of resistance training on HGS and TUG, two studies had different results. And as there are not enough data for a meta-analysis of these two outcomes, no definitive conclusions can yet be drawn.

Although our study showed that resistance training can increase BMD, of the 4 studies included in the meta-analysis for BMD, only Li et al. demonstrated a significant increase in BMD [52]; the other three studies showed no significant difference between the experimental and control groups. In addition, it is worth noting that the intervention method in the study by Li et al. [52] was resistance training combined with aerobic exercise, so it is impossible to determine how much benefit of its influence on BMD was derived from resistance training; therefore, we should be cautious when interpreting this result. We noted that the merged data result of BFP had moderate heterogeneity (*I*^2^ = 50%). Through sensitivity analysis, we found that the source of heterogeneity was the study conducted by Banitalebi et al. [53], and the heterogeneity may be a result of their definition of obesity and the characteristics of the participants. Overall, the four randomized controlled studies included in this study had a low risk of bias, the GRADE evidence for outcomes was rated as low (four outcomes) and moderate (five outcomes), and only one outcome was moderately heterogeneous and acceptable. Therefore, our conclusions may provide some reference for the development of clinical guidelines.

Osteoporosis, sarcopenia, and obesity were once considered separate diseases and were rarely studied together. Later, people combined two of these conditions, sarcopenia and obesity [59, 60], as well as osteoporosis and sarcopenia [61, 62], and explored them. OSO is a syndrome of osteoporosis, sarcopenia, and obesity that was first proposed in 2014 and indicates a link between bone, muscle, and fat [30]. The diagnosis of OSO should be carried out from two aspects: physical via body composition measurements and functional via physical performance measures [31]. Body composition can be measured in three ways: BMD *T*-score to check for osteoporosis, appendicular lean mass to identify sarcopenia, and BFP to determine obesity [31]. At present, the assessment tools for body composition mainly include DXA and BIA. Physical performance tests, such as HGS, single leg stance, and GS, could be assessed with minimal equipment [51].

The safety of the intervention is a key issue that researchers must consider. With the exception of one study that reported that a small number of patients in the experimental group reported muscle soreness, knee pain, and shoulder pain (25%) during the first three sessions of training [53], no other studies reported adverse events, suggesting that resistance training is a safe intervention for older adults.

To our knowledge, this is the first systematic review and meta-analysis of the effects of resistance training on elderly patients with OSO. The results of this study indicate that resistance training has a significant beneficial effect in the treatment of OSO in many aspects, and this effect is affected by the intensity of resistance training; the higher the intensity is, the greater the effect. Nevertheless, in one of the included studies, the intervention method combined aerobic exercise with resistance training [52], so we cannot be sure how much of the final intervention benefit was due to resistance training, which may be a source of bias.

In addition, this study had some limitations. First, the number of studies included in this analysis was very limited, with only four small samples, which may have some impact on the accuracy of the outcomes. Second, the intervention time in all four studies was short, at just 12 weeks. Therefore, future studies with large samples and long intervention durations are needed to verify these results.

In summary, resistance training is a safe and effective intervention for elderly patients with OSO. The government should vigorously promote resistance training, and the public should also actively respond, which may have a good effect on the prevention of OSO. Resistance training may also reduce some of the economic burden resulting from OSO.

## Conclusions

Resistance training is a safe and effective intervention that can improve many parameters in OSO patients, such as BFP and SMM. Resistance training is a good option for elderly individuals who want to improve their physical fitness.

## Supplementary Information

Below is the link to the electronic supplementary material.

Supplementary file1 (DOCX 45 KB)

## Data Availability

Published data used for the systematic review and meta-analysis are available from the authors.
