# Supplementary material for: Effects of resistance training on body composition and physical function in elderly patients with osteosarcopenic obesity: a systematic review and meta-analysis
Source: Arch Osteoporos. 2022 Jun 3;17(1):82. doi: 10.1007/s11657-022-01120-x (PMC9163017; doi:10.1007/s11657-022-01120-x)
Supplement: Supplementary file 1 — Supplementary file1 (DOCX 45 KB) [file 11657_2022_1120_MOESM1_ESM.docx]

**Appendices:**

**Supplemental Material for *Effects of resistance training on body composition and physical function in elderly patients with osteosarcopenic obesity: a systematic review and meta-analysis.***

**Table S1.** The PubMed search strategy.

**Table S2.** List of excluded literature and reasons for exclusion.

**Table S3.** Resistance training protocol of each study.

**Table S4.** The Physiotherapy Evidence Database (PEDro) score of each study.

**Table S1**. The PubMed search strategy.

| Database | PubMed |
| --- | --- |
| Steps  #1  #2  #3  #4  #5 | **Search terms for query**  osteosarcopeni* or osteo-sarcopeni* or sarco-osteopeni* or sarco-osteoporo*  obesity or obese or adiposity or overweight  resistance training or resistance exercise or strength training  randomized controlled or RCT or controlled trial or clinical trial  #1 and #2 and #3 and #4 |

**Table S2.** List of excluded literature and reasons for exclusion (21 studies).

| Study | Title | Reasons for exclusion |
| --- | --- | --- |
| Abdelhafiz 2021  Agostini 2021  Banitalebi 2021  Cunha 2020  Cunha 2021  Santos 2020  Gandham 2021  Guest 2021  Dayani 2018  Banitalebi 2019  Karava 2021  Kazemi 2021  Kemmler 2020  Kemmler 2020  Kemmler 2020  Kemmler 2021  Poggiogalle 2021  Salvadori 2021  Stengel 2021  Wen 2021  Wijayatunga 2021 | Impact of frailty metabolic phenotypes on the management of older people with type 2 diabetes mellitus  Rehabilitative Good Practices in the Treatment of Sarcopenia: A Narrative Review  Effect of 12-weeks elastic band resistance training on MyomiRs and osteoporosis markers in elderly women with Osteosarcopenic obesity: a randomized controlled trial  Resistance Training Performed with Single and Multiple Sets Induces Similar Improvements in Muscular Strength, Muscle Mass, Muscle Quality, and IGF-1 in Older Women: A Randomized Controlled Trial  Comparision of Low and High Volume of Resistance Training on Body Fat and Blood Biomarkers in Untrained Older Women: A Randomized Clinical Trial  Physical activity is associated with functional capacity of older women with osteosarcopenic obesity: 24-month prospective study  Falls, fractures, and areal bone mineral density in older adults with sarcopenic obesity: A systematic review and meta-analysis  New Therapeutic Approaches and Biomarkers for Increased Healthspan  Effect of Elastic Resistance Band Training on Biomarkers of Osteosarcopenia obesity  Effect of resistance training on growth and osteogenic factors of elderly women  Muscle-bone axis in children with chronic kidney disease: current knowledge and future perspectives  Obesity, but not hyperandrogenism or insulin resistance, predicts skeletal muscle mass in reproductive-aged women with polycystic ovary syndrome: A systematic review and meta-analysis of 45 observational studies  Effects of High-Intensity Resistance Training on Fitness and Fatness in Older Men with Osteosarcopenia  Effects of High-Intensity Resistance Training on Osteopenia and Sarcopenia Parameters in Older Men with Osteosarcopenia-One-Year Results of the Randomized Controlled Franconian Osteopenia and Sarcopenia Trial (FrOST)  Effect of high-intensity resistance exercise on cardiometabolic health in older men with osteosarcopenia: the randomised controlled Franconian Osteopenia and Sarcopenia Trial (FrOST)  Changes in Body Composition and Cardiometabolic Health After Detraining in Older Men with Osteosarcopenia: 6-Month Follow-Up of the Randomized Controlled Franconian Osteopenia and Sarcopenia Trial (FrOST) Study  Therapeutic strategies for sarcopenic obesity: a systematic review  Playing around the anaerobic threshold during COVID-19 pandemic: advantages and disadvantages of adding bouts of anaerobic work to aerobic activity in physical treatment of individuals with obesity  Effects of 16 months of high intensity resistance training on thigh muscle fat infiltration in elderly men with osteosarcopenia  Resistance exercise affects catheter-related thrombosis in rats through miR-92a-3p, oxidative stress and the MAPK/NF-κB pathway  Normal weight obesity and unaddressed cardiometabolic health risk-a narrative review | Not osteosarcopenic obesity  Not osteosarcopenic obesity  Repeated primary outcome  Not osteosarcopenic obesity  Not osteosarcopenic obesity  Not randomized controlled trial  Not osteosarcopenic obesity  Not osteosarcopenic obesity  Full text unavailable  Not osteosarcopenic obesity  Not osteosarcopenic obesity  Not osteosarcopenic obesity  Not osteosarcopenic obesity  Not osteosarcopenic obesity  Not osteosarcopenic obesity  Not osteosarcopenic obesity  Not osteosarcopenic obesity  Not osteosarcopenic obesity  Not osteosarcopenic obesity  Not osteosarcopenic obesity  Not osteosarcopenic obesity |

**Table S3.** Resistance training protocol of each study.

| Study | Means of intervention | Description of interventions | Time  (min) | Frequency  (times/week) | Period  (weeks) |
| --- | --- | --- | --- | --- | --- |
| Banitalebi et al. [[54](#_ENREF_54)]  Lee et al. [[52](#_ENREF_52)]  Li et al. [[53](#_ENREF_53)]  Cunha et al. [[51](#_ENREF_51)] | EBRT  peRET  AE+RT  RT | 10 min of warm-up exercises + 60 min EBRT incorporating one to two exercises (in a slow controlled manner, 2 s for concentric phase and 4 s for eccentric phase) + cool-down routine. The EBRT was designed to train all major muscle groups (namely; legs, back, abdomen, chest, shoulder, and arms). After 4 weeks of low resistance training, resistance levels gradually increased.  10 min of warm-up exercises + 40 min EBRT + 5 min of cooling-down exercises. The resistance exercises targeted all major muscle groups in the shoulders, arms, lower limbs, chest, and abdomen, with 1–2 exercises included for each muscle group. A total of 3 sets of 10 repetitions of gentle concentric and eccentric contractions through the full range of motion were performed for each exercise. Resistance levels from low to high  AE: Moderate intensity brisk walking at a pace of 90-130 steps/min;  RT: Multiple major muscle groups of limbs and trunk were used for resistance strength training. The exercise intensity was controlled by elastic band resistance control and Borg subjective strength scale. Strength training started at low doses, with 1 set of 10 reps in week 1, another set in week 2, and 3 sets in weeks 3-4. Rest 1-3 min between each group.  G1S performed 1 set of 10–15 repetitions maximum for each exercise.  G3S performed 3 sets of 10–15 repetitions maximum for each exercise (1-2 min rest interval between sets).  Progression was planned when the upper limits of the repetitions-zone were completed for two consecutive training sessions; then weight was increased 2–5% for the upper limb exercises and 5–10% for the lower limb exercises to the next session. | 60  40  AE: 30~45  RT: 45~60  G1S: 30  G3S: 50 | 3  3  AE: 5  RT: 3  3 | 12  12  12  12 |

**Abbreviations:** EBRT: elastic band resistance training; AE: aerobic exercise; RT: resistance training; peRET: progressive elastic band resistance exercise training; G1S: 1 set group; G3S: 3 sets group.

**Table S4**. The Physiotherapy Evidence Database (PEDro) score of each study.

| Study | (1) | (2) | (3) | (4) | (5) | (6) | (7) | (8) | (9) | (10) | (11) | Score |
| --- | --- | --- | --- | --- | --- | --- | --- | --- | --- | --- | --- | --- |
| Cunha et al. [[51](#_ENREF_51)]  Banitalebi et al. [[54](#_ENREF_54)]  Lee et al. [[52](#_ENREF_52)]  Li et al. [[53](#_ENREF_53)] | Yes  Yes  Yes  Yes | 1  1  1  1 | 1  1  1  0 | 1  1  1  1 | 0  0  0  0 | 0  0  0  0 | 1  1  1  0 | 1  0  1  1 | 0  1  1  0 | 1  1  1  1 | 1  1  1  1 | 7  7  8  5 |

**Notes**: Yes, one point; No, score 0. A total PEDro score is achieved by adding the ratings of (2) to (11) for a combined total score between 0 to 10.

(1) eligibility criteria were specified, (2) random allocation, (3) concealed allocation, (4) baseline comparability, (5) participant blinding, (6) therapist blinding, (7) assessor blinding, (8) adequate follow-up (> 85%), (9) intention-to-treat analysis, (10) between-group statistical comparisons, and (11) point and variability measurements.
